# Safety evaluation and toxicokinetics of repeated inhalation exposure to pegylated interferon α-2b

**DOI:** 10.3389/fphar.2025.1689087

**Published:** 2025-12-11

**Authors:** Cuifang Ji, Xunlian Ji, Huan Li, Qinfang Wang

**Affiliations:** 1 Department of Pharmacy, Haikou Affiliated Hospital of Central South University Xiangya School of Medicine, Haikou, Hainan, China; 2 Department of Infectious Diseases, Shanxi Children’s Hospital (Shanxi Women and Children Hospital), Taiyuan, Shanxi, China

**Keywords:** pegylated interferon α-2b, inhalation toxicology, juvenile animal study, toxicokinetics, NOAEL

## Abstract

This study aimed to evaluate the safety profile and toxicokinetic (TK) characteristics of pegylated interferon α-2b (PEG IFNα-2b) following repeated nebulized inhalation exposure in juvenile Sprague-Dawley (SD) rats, and to establish the no-observed-adverse-effect level (NOAEL) to support its clinical development for pediatric respiratory infections. A total of 190 juvenile SD rats (5–6 days old) were randomized into blank control, low- (126.4 ± 21.8 μg/kg), mid- (384.3 ± 66.9 μg/kg), and high-dose (1,202.4 ± 176.4 μg/kg) groups. Animals received nebulized PEG IFNα-2b *via* whole-body/nasal inhalation once every 2 days for two consecutive weeks. Assessments included clinical observations, body weight, hematology, coagulation, serum biochemistry, developmental parameters, histopathology, and TK analysis. PEG IFNα-2b was well tolerated at all dose levels, with no treatment-related mortality, clinical signs, or histopathological alterations observed. Systemic exposure increased in a dose-proportional manner; concentrations in the low-dose group were below the lower limit of quantitation (LLOQ), while C_max_ values ranging from 0.467 ng/mL (mid-dose, females) to 15.3 ng/mL (high dose, females), and AUC_0-last_ values from 2.18 h·ng/mL (mid-dose, females) to 61.5 h·ng/mL (high dose, females). Accumulation factors ranged from 0.135 to 1.66, indicating no significant drug accumulation. Developmental parameters and histopathological examinations of respiratory tissues revealed no treatment-related abnormalities. The NOAEL was determined to be 1,202.4 ± 176.4 μg/kg, the highest dose tested. Inhaled PEG IFNα-2b demonstrated a favorable safety and TK profile in juvenile rats at doses up to 1,202.4 ± 176.4 μg/kg, supporting its potential as an inhaled antiviral therapy for pediatric respiratory infections.

## 1 Introduction

Respiratory syncytial virus (RSV) is the leading cause of acute lower respiratory tract infections (LRTIs) in infants and young children worldwide, accounting for approximately 33 million cases annually and an estimated 101,400 deaths in children under 5 years of age, with more than 97% of fatalities occurring in low-income countries (Li et al., 2022). In China, RSV infections result in approximately 950,000 hospitalizations each year among children under five, placing the country among those with the highest global burden of RSV-associated pediatric LRTIs (Li et al., 2021).

RSV infection typically presents with mild upper respiratory symptoms, such as cough and rhinorrhea, but can rapidly progress to severe complications, including bronchiolitis, pneumonia, and hypoxemia, particularly in infants (Kaler et al., 2023). Current management strategies remain largely supportive, focusing on airway clearance, oxygen supplementation, and hydration (Dalziel et al., 2022). Although nebulized 3% hypertonic saline has shown potential for symptom relief and reduction in hospitalization duration, inconsistent clinical evidence has limited its routine use (Brooks et al., 2016; Angoulvant et al., 2017). Furthermore, international guidelines discourage the use of nebulized or systemic bronchodilators, glucocorticoids, and antibiotics due to a lack of demonstrated efficacy (Dalziel et al., 2022; Zhang et al., 2024). These limitations underscore the urgent need for safer and more effective antiviral therapies, especially in light of the toxicity concerns associated with current treatments, such as ribavirin—a nucleoside analog known for its teratogenic risks to healthcare workers upon aerosol exposure and its potential to induce hematotoxicity in patients (Tejada et al., 2022).

Interferon-alpha (IFNα), a type I interferon, is a critical effector cytokine in antiviral immunity, exhibiting both antiviral and immunomodulatory properties (González-Navajas et al., 2012). Since the 1990s, recombinant human IFNα (rhIFNα) has been explored as a therapeutic option for pediatric RSV infection. However, conventional subcutaneous administration often results in systemic overexposure and suboptimal clinical efficacy (Chipps et al., 1993). Recent advances in inhalation drug delivery have reignited interest in IFNα-based therapies for viral LRTIs. Preclinical studies have demonstrated that nebulized IFNα can achieve high lung tissue concentrations while limiting systemic exposure (Liu et al., 2013). Clinical trials have further confirmed its ability to alleviate lower respiratory symptoms, reduce hospitalization time, and maintain an acceptable safety profile in pediatric patients with viral pneumonia (Chen et al., 2020; Jiang et al., 2020).

Despite these promising results, rhIFNα as a protein-based therapeutic faces key limitations in the treatment of pulmonary diseases, including rapid pulmonary degradation and clearance, which necessitate frequent dosing due to its short half-life (Mcleod et al., 2015). PEGylation, a well-established strategy for extending the half-life of biopharmaceuticals, can mitigate these drawbacks by enhancing pulmonary retention, improving chemical stability during nebulization, and potentially reducing the risk of pulmonary immunogenicity (Mcleod et al., 2015).

PEG IFNα-2b (Pegbing®, Xiamen Amoytop Biotech Co., Ltd.) is a long-acting conjugate of recombinant human IFNα-2b and a 40 kDa Y-shaped branched polyethylene glycol (PEG) moiety (Hou et al., 2017). In September 2016, it was approved by the National Medical Products Administration (NMPA) of China for the subcutaneous treatment of chronic hepatitis B and C. Clinical data have demonstrated that long-term PEG IFNα-2b administration in patients with chronic hepatitis exhibits an acceptable safety profile, with adverse effects primarily limited to transient flu-like symptoms (Wu et al., 2021; Wang et al., 2025). Notably, *in vitro* studies evaluating antiviral efficacy against RSV revealed PEG IFNα-2b’s superior stability and potency compared to unmodified IFNα-2b (Liu and Dai, 2025). Additionally, unpublished preclinical data from New Zealand White rabbits showed that nebulized PEG IFNα-2b achieved a tenfold higher pulmonary exposure compared to unmodified IFNα-2b at 48 h post-dose, with detectable lung concentrations persisting up to 72 h—a time point at which unmodified IFNα-2b was no longer detectable. A recent phase 1 randomized trial demonstrated that single-dose nebulized PEG IFNα-2b (90 and 180 μg) was well-tolerated in healthy adults, with only mild hematological adverse events and minimal systemic absorption. Pharmacodynamic analysis revealed dose-dependent increases in neopterin levels, though these remained below thresholds associated with systemic immune activation (Huang et al., 2025). These findings suggest that PEG IFNα-2b may enhance therapeutic outcomes through its favorable safety profile while improving local drug retention, reducing dosing frequency, and increasing patient compliance.

In this study, we systematically evaluated the safety and toxicokinetics of repeated nebulized PEG IFNα-2b administration in juvenile Sprague-Dawley (SD) rats under Good Laboratory Practice (GLP)-compliant conditions. Through TK analysis, longitudinal growth assessment, and histopathological examination, we aimed to establish the no-observed-adverse-effect level (NOAEL) to inform clinical trial design for this promising RSV therapeutic candidate.

## 2 Materials and methods

### 2.1 Experimental animals and ethics

SPF-grade SD rats (5–6 days old; males, 11–15 g; females, 11–14 g) were obtained from Zhejiang Vital River Laboratories. Animals were housed under controlled environmental conditions (temperature: 21 °C–26 °C; humidity: 30%–70%; light/dark cycle: 12 h) with *ad libitum* access to sterilized feed and water. Dams and offspring were maintained in polycarbonate cages (10 pups per dam) until weaning. All procedures were approved by the Institutional Animal Care and Use Committee (IACUC No. S-ACU24-1750) in accordance with AAALAC guidelines. At the end of the study, euthanasia was performed under deep anesthesia induced by isoflurane (Beiyining®, 100 mL, Orbiepharm, China). Specifically, 1–10 mL of liquid isoflurane was introduced into a sealed chamber containing absorbent cotton at the bottom. Rats were placed in the chamber until loss of consciousness (approximately 1 min), followed by exsanguination *via* the abdominal aorta.

### 2.2 Aerosol generation and characterization

#### 2.2.1 Aerosol generation system

Aerosols of PEG IFNα-2b (Pegbing®, 180 μg/0.5 mL; Xiamen Amoytop Biotech Co., Ltd.) were generated using a PARI LC SPRINT jet nebulizer (PARI GmbH, Germany) operated at a flow rate of 7 L/min. The nebulizer was calibrated to deliver the drug at a rate of 0.25–1.0 mL/min. The resulting aerosol was introduced into a whole-body exposure chamber (HRH-MNE1628, Beijing Huironghe Technology Co., Ltd., China) under controlled environmental conditions (temperature: 20 °C–26 °C; relative humidity: 30%–80%; O_2_ ≥ 19%; CO_2_ ≤ 1%).

#### 2.2.2 Particle size distribution analysis

Aerodynamic properties of the aerosol were characterized using a Next-Generation Impactor (NGI-1342 and NGI-1872, Copley Scientific, UK) operated at a flow rate of 15.24–15.33 L/min. Aerosol samples were collected over 5 min on 75 mm glass fiber filters, and aerosol concentrations at each NGI stage were quantified *via* a validated high performance liquid chromatography (HPLC) method. The HPLC analysis employed a Jupiter® C18 column (4.6 × 250 mm, 5 μm; Phenomenex, Inc.) with a binary mobile phase consisting of 0.1% trifluoroacetic acid in water (A) and 0.1% trifluoroacetic acid in acetonitrile (B). Gradient elution was programmed as follows: 0 min, 30% B; 6 min, 80% B; 6.01 min, 30% B (total run time: 11 min). The flow rate was 1.0 mL/min, column temperature maintained at 30 °C, and detection performed at 210 nm with an injection volume of 50 μL.

Mass median aerodynamic diameter (MMAD), geometric standard deviation (GSD), and fine particle fraction (FPF, percentage of particles with aerodynamic diameter < 5 μm) were calculated using Copley Inhaler Testing Data Analysis Software (v3.10).

#### 2.2.3 Pulmonary deposition kinetics

Pulmonary deposition kinetics were estimated based on species-specific respiratory parameters. Respiratory minute volume (RMV) was calculated using the equation (Alexander et al., 2008):

$$RMV\left(L/\min\right)=0.608\times {\text{BW}\left(\text{kg}\right)}^{0.852}$$

The delivered dose (μg/kg) was determined using the formula:

Dose=RMV×C×t/BW

where C is the aerosol concentration (μg/L), t is the exposure duration (min), and BW is the body weight (kg).

### 2.3 Dose groups and exposure regimens

A total of 190 juvenile SD rats were allocated into eight groups (Table 1), with animals of both sexes equally distributed across the main study groups (n = 6/sex/group) and the satellite groups designated for TK analysis (n = 14–19/sex/group), using body weight-stratified randomization. The selection of target dose levels—140 μg/kg (low), 453 μg/kg (mid), and 1,360 μg/kg (high)—was informed by aerosol delivery parameters established in a preceding feasibility study. These doses were chosen to establish a clinically relevant safety margin. The pulmonary deposition dose, calculated as the product of the delivered dose and the deposition fraction, is considered more indicative of the actual effective dose for inhaled drugs. Based on guidance from the U.S. Food and Drug Administration (FDA), the deposition fractions for rats and humans are estimated to be 10% and 100%, respectively (Jones and Baldrick, 2013). Therefore, the target delivered doses of 140, 453, and 1,360 μg/kg in rats correspond to estimated pulmonary deposition doses of approximately 14, 45.3, and 136 μg/kg. The proposed clinical dose for children is 45 or 90 µg/dose, which, assuming a body weight of 10 kg, translates to a lung deposition dose of 4.5 or 9 μg/kg. Accordingly, the low dose in this study provides a safety margin of ≥1.5-fold and the high dose ≥ 15-fold relative to the anticipated clinical lung dose.

**TABLE 1:** Experimental design and dose groups.

Group		Dose level	Target dose (μg/kg)	Inhalation duration (min)	Number of animals
Main study groups	1	Control	0	240 (D1–D7)	3M + 3F
				180 (D8–D13)	
	2	Low-dose	140	25 (D1–D7)	3M + 3F
				20 (D8–D13)	
	3	Mid-dose	453	80 (D1–D7)	3M + 3F
				60 (D8–D13)	
	4	High-dose	1,360	240 (D1–D7)	3M + 3F
				180 (D8–D13)	
Satellite groups	5	Control	0	240 (D1–D7)	14M + 14F
				180 (D8–D13)	
	6	Low-dose	140	25 (D1–D7)	19M + 19F
				20 (D8–D13)	
	7	Mid-dose	453	80 (D1–D7)	19M + 19F
				60 (D8–D13)	
	8	High-dose	1,360	240 (D1–D7)	19M + 19F
				180 (D8–D13)	

M, male; F, female.

In the preceding feasibility study, PEG IFNα-2b aerosols were generated with a mean concentration of 7.2 μg/L, enabling a projected theoretical delivered dose of 1824 μg/kg following 4 h of continuous inhalation. To ensure proportional dose escalation and procedural feasibility in juvenile animals, the low-dose group received 25-min exposures during Days 1–7 and 20-min exposures during Days 8–13; the mid-dose group received 80-min and 60-min exposures; and the high-dose group received 240-min and 180-min exposures for the respective periods (Table 1). This progressive reduction in exposure duration from Days 1–7 to Days 8–13 accommodated age-dependent increases in RMV, thereby maintaining consistent delivered doses per kilogram body weight throughout the study. The high-dose regimen represented the maximal tolerable exposure duration deemed practical for this age group, ensuring adequate safety margin assessment.

The main study groups were subjected to comprehensive safety assessments, while satellite groups were designated for TK analysis. PEG IFNα-2b was administered once every 2 days over a 2-week period (total of seven doses), with the first administration defined as Day 1 (D1).

### 2.4 Safety profile assessment

#### 2.4.1 Clinical observations and developmental parameters

All animals were monitored at least twice daily (morning and afternoon) throughout the study to assess mortality and potential treatment-related clinical signs. Comprehensive evaluations—including assessments of integumentary and mucosal integrity, neurological and behavioral activity, respiratory function, gastrointestinal signs, and urogenital abnormalities—were performed on the main study groups at baseline and weekly following drug administration.

Body weights were recorded at baseline and twice weekly during the dosing period for the main study groups (six animals per sex per group), with final weights measured prior to scheduled euthanasia. Developmental parameters—including tibial length (measured from the knee to the ankle of the right hind limb), body length (crown-to-rump), and tail length (rump-to-tip)—were assessed at baseline and weekly during the dosing phase in the main study groups (three animals per sex per group). Measurements were conducted using digital calipers to ensure accuracy.

#### 2.4.2 Hematological evaluation

Blood samples were collected *via* the abdominal aorta from the main study groups on D14 prior to planned euthanasia for comprehensive hematological, biochemical, and coagulation analyses.

Serum biochemical parameters were analyzed using a Beckman Coulter AU5800 automated analyzer (Beckman Coulter, USA). Markers of hepatic function included aspartate aminotransferase (AST), alanine aminotransferase (ALT), total protein (TP), albumin (Alb), albumin-to-globulin ratio (A/G), direct bilirubin (DBIL), total bilirubin (TBIL), alkaline phosphatase (ALP), creatine kinase (CK), and γ-glutamyl transferase (GGT). Renal function was evaluated by measuring urea (UREA) and creatinine (CRE). Additional parameters included lipid profile [total cholesterol (TC), triglycerides (TG)], glucose (Glu), and electrolytes [calcium (Ca), phosphorus (P), sodium (Na^+^), potassium (K^+^), chloride (Cl^−^)].

Hematological profiles were assessed using a Sysmex XN-9000 automated hematology analyzer (Sysmex Corporation, Japan). Evaluated indices included red blood cell count (RBC), hemoglobin (HGB), hematocrit (HCT), mean corpuscular volume (MCV), mean corpuscular hemoglobin (MCH), mean corpuscular hemoglobin concentration (MCHC), reticulocyte count (RET), white blood cell count (WBC) and differential (LYMPH%, MONO%, EOS%, BASO%, NEUT%), and platelet count (PLT).

Coagulation parameters—prothrombin time (PT), activated partial thromboplastin time (APTT), and fibrinogen (FIB)—were measured using a Sysmex CS-5100 coagulation analyzer (Sysmex Corporation, Japan).

#### 2.4.3 Histological observations

Full necropsies were conducted on D14 for all animals in the main study groups following euthanasia. Eyes, testes, and Harderian glands were fixed in Davidson’s solution, while remaining organs—including respiratory tissues (nasal turbinate, larynx, lungs with bronchi, bronchial lymph nodes, tongue, and trachea)—were immersion-fixed in 10% neutral buffered formalin. Histopathological evaluations were conducted on hematoxylin and eosin (H&E)-stained sections of respiratory tissues and any gross lesions observed during necropsy.

### 2.5 Toxicokinetic assessment

Serial blood samples (0.3 mL per animal) were collected from satellite groups under isoflurane anesthesia *via* the abdominal aorta on D1 or the jugular vein on D13 at predefined timepoints. For Groups 6–8, samples were collected at pre-dose, 5 min, 30 min, 1.5 h, and 8 h post-exposure. For Group 5, sampling occurred at pre-dose and 1.5 h post-exposure. Each timepoint included two animals per sex.

Plasma concentrations of PEG IFNα-2b were quantified using a validated liquid chromatography–tandem mass spectrometry (LC-MS/MS) method, with a lower limit of quantification (LLOQ) of 0.300 ng/mL. Pharmacokinetic parameters were derived using non-compartmental analysis (WinNonlin, version 8.3.5). Parameters included maximum observed serum concentration (C_max_), time to reach C_max_ (T_max_), and the area under the concentration-time curve from time zero to the last measurable timepoint (AUC_0-last_). Dose proportionality was evaluated by comparing dose-normalized AUC_0-last_ values across groups. Drug accumulation was assessed by calculating accumulation factors (AF), defined as the ratio of AUC_0-last_ on D13 to that on D1 (AF = AUC_0-last,D13_/AUC_0-last,D1_).

### 2.6 Statistical analysis

All statistical analyses were performed using two-tailed tests with a significance threshold set at P ≤ 0.05. Data are expressed as mean ± standard deviation (SD) and were analyzed using Provantis® software (SAS 9.4-based platform). Graphs were generated using GraphPad Prism version 10.4.2 (GraphPad Software, USA). Comparisons between treatment and control groups were stratified by sex. Animals lacking unilateral paired organs were excluded from organ weight analysis.

Levene’s test was applied to assess variance homogeneity. For datasets demonstrating homogeneity (P > 0.05), one-way analysis of variance (ANOVA) was conducted followed by Dunnett’s *post hoc* test for multiple comparisons. For heteroscedastic data (P ≤ 0.05), data were either log-transformed (with zero values replaced by one-tenth of the minimum non-zero value) or subjected to non-parametric analysis using the Kruskal-Wallis test, followed by Mann-Whitney U test and Bonferroni correction for multiple comparisons.

## 3 Results

### 3.1 Aerosol characterization and pulmonary deposition

The PEG IFNα-2b aerosol demonstrated a MMAD ranging from 2.1 to 2.5 μm across all dose groups, with a FPF consistently exceeding 90%. Although the actual delivered doses (low: 126.4 ± 21.8 μg/kg; mid: 384.3 ± 66.9 μg/kg; high: 1,202.4 ± 176.4 μg/kg) deviated from target values by <15%—primarily attributable to inherent aerosol concentration fluctuations and inter-animal respiratory heterogeneity—this variation level demonstrates high dosing precision and consistent aerosol generation (Table 2).

**TABLE 2:** Delivered dose and particle size characteristics.

Parameter	Low-dose group	Mid-dose group	High-dose group
Target dose (μg/kg)	140	453	1,360
Actual dose (μg/kg)	126.365 ± 21.796	384.265 ± 66.983	1,202.357 ± 176.371
Mean MMAD (μm)	2.158 ± 0.411	2.128 ± 0.240	2.533 ± 0.043
Mean GSD	1.529 ± 0.230	1.614 ± 0.068	1.664 ± 0.034
Mean FPF (%)	95.254 ± 4.196	96.379 ± 3.269	91.098 ± 0.860

MMAD, mass median aerodynamic diameter; GSD, geometric standard deviation; FPF, fine particle fraction. Data are shown as Mean ± SD.

### 3.2 General safety and developmental parameters

No treatment-related mortality or moribundity was observed in any dose group throughout the study period. Clinical examinations revealed no adverse signs in animals from the low-, mid-, or high-dose groups.

**FIGURE 1:**
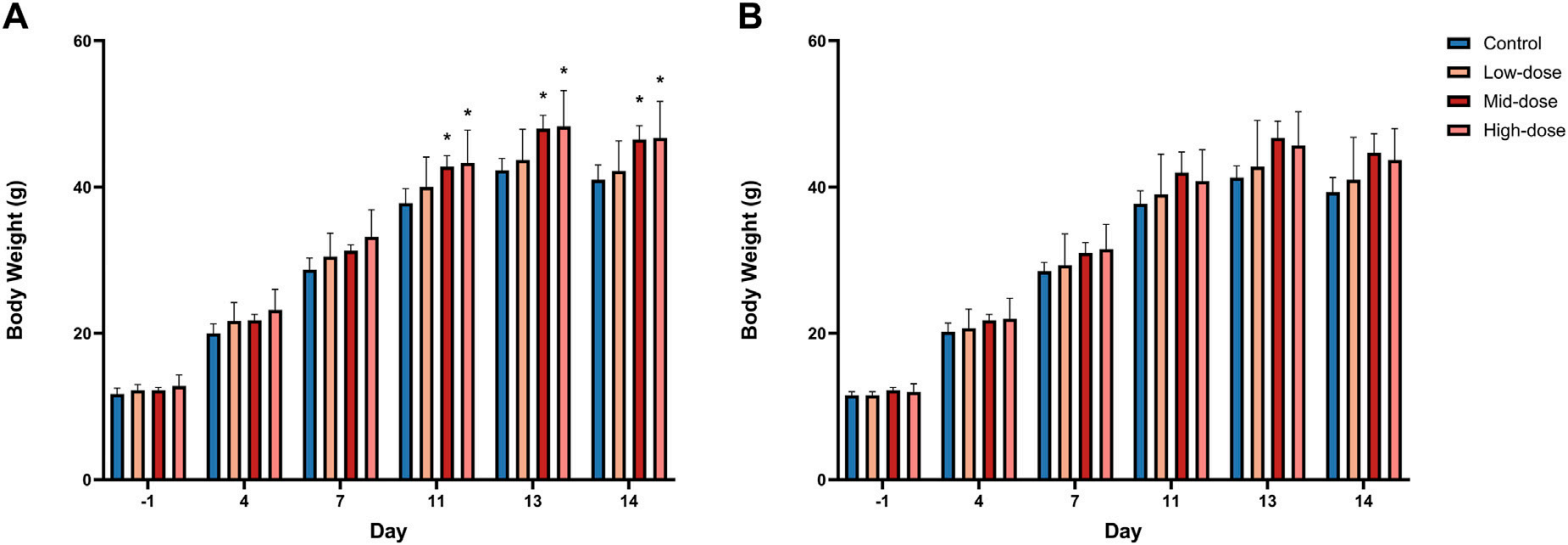
Body Weight Trends in Rats (n = 6/group/sex). **(A)** male, **(B)** female. Data are shown as Mean ± SD. *P ≤ 0.05 vs. control by Kruskal-Wallis and Wilcoxon test.

Body weight trajectories were generally comparable to controls. Notably, male animals in the mid- and high-dose groups exhibited statistically significant increases in body weight (P ≤ 0.05; Figure 1; individual data shown in Supplementary Figure S1; Supplementary Table S1). However, these changes were considered toxicologically insignificant due to the absence of a clear dose-response relationship, confinement to a single sex (males only), the nature of the effect (increased weight rather than weight loss), and their full alignment with the normal growth range of male juvenile SD rats of the same age under identical housing conditions—none of which support biological plausibility for toxicity.

**FIGURE 2:**
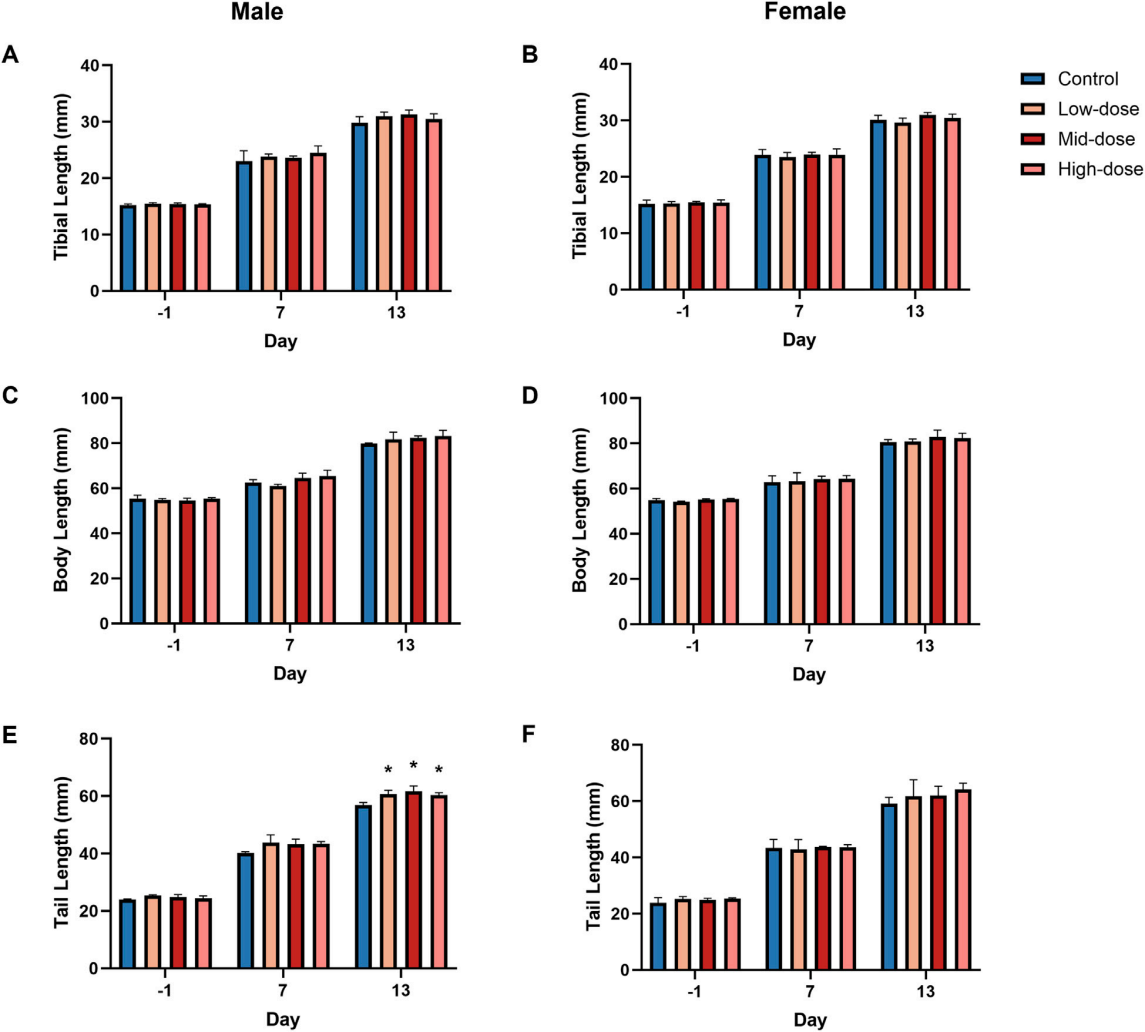
Developmental Parameter Changes in Rats (n = 3/group/sex). **(A,B)** tibial length; **(C,D)** body length; **(E,F)** tail length. Data are shown as Mean ± SD. *P ≤ 0.05 vs. control by ANOVA and Dunnett test.

Developmental parameters (tibia length, body length, and tail length) were generally comparable to those of the control group (Figure 2; individual data shown in Supplementary Figures S2–S4, respectively). A slight increase in tail length was observed in male animals across all dose groups on D14 (low-dose: +6.8%, mid-dose: +8.5%, high-dose: +6.1%). However, the lack of a clear dose-response relationship and the small magnitude of change indicate that these differences are not biologically meaningful.

### 3.3 Hematology, biochemistry, and histopathology

Hematological assessments revealed no treatment-related alterations in erythrocyte, leukocyte, or platelet indices (Supplementary Table S5). A statistically significant increase in neutrophil percentage (NEUT%: +9.4%, P ≤ 0.05) was observed in high-dose males on D14; however, absolute neutrophil counts remained unchanged and no associated pathological or clinical findings were identified, suggesting a lack of toxicological significance.

Coagulation parameters (Supplementary Table S6) and serum biochemistry profiles (Supplementary Table S7) exhibited no consistent dose-dependent changes. Isolated decreases in GGT (−18.2%) and Glu (−12.5%) were noted in low-dose males at D14, but the absence of a dose-response trend and minimal magnitude of change support their classification as incidental findings.

**FIGURE 3:**
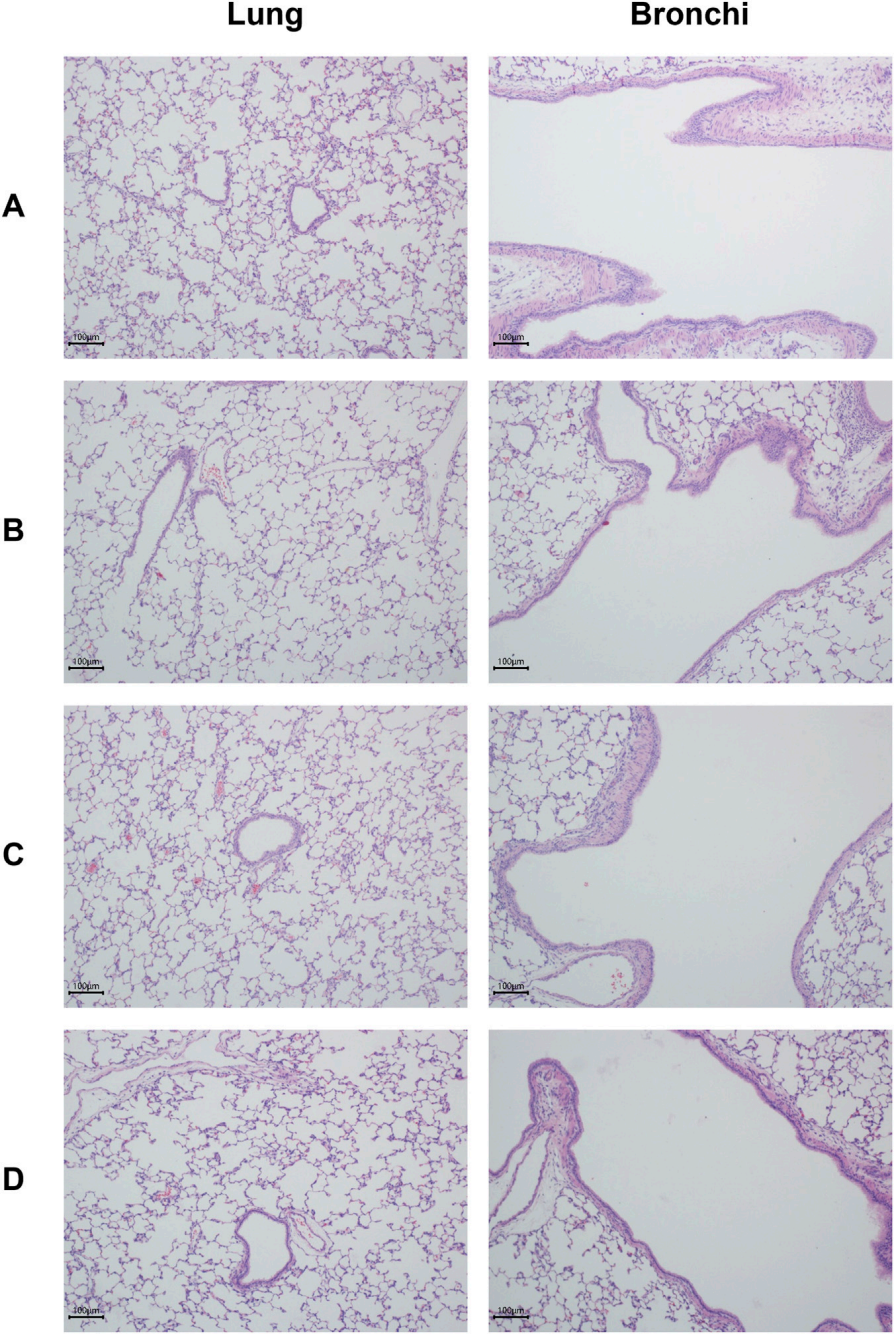
Histopathological Examination of Lungs and Bronchi in Rats on Day 14. Hematoxylin and eosin (H&E)-stained sections of lung and bronchial tissues. **(A)** control group; **(B)** low-dose group; **(C)** mid-dose group; **(D)** high-dose group. All tissues exhibit normal histological architecture. Magnification, ×100; scale bar = 100 μm.

Histopathological examination of respiratory tissues—including the nasal cavity, larynx, trachea, lungs, and bronchial lymph nodes—revealed no treatment-related lesions in any dose group (Figure 3).

### 3.4 Toxicokinetic profile

Serum concentrations of PEG IFNα-2b in control and low-dose groups remained below the LLOQ (0.300 ng/mL) throughout the study. Dose-dependent increases in both C_max_ and AUC_0-last_ were observed across treatment groups, with notable sex-based differences in systemic exposure (Table 3).

**TABLE 3:** Toxicokinetic parameters of PEG IFNα-2b in serum from mid- and high-dose groups.

Cycle	Dose	Sex	T_max_ (h)	C_max_ (ng/mL)	C_max_ ratio (High-/Mid-)	AUC_0-last_ (h·ng/mL)	AUC_0-last_ ratio (High-/Mid-)	AF
D1	Mid-dose	M	1.42	0.623	M: 1.59	5.02	M: 1.49	—
		F	9.33	0.467		2.18		—
	High-dose	M	12.0	0.990	F: 32.72	7.50	F: 28.23	—
		F	5.50	15.3		61.5		—
D13	Mid-dose	M	0.00	0.357	M: 1.84	0.838	M: 7.01	0.167
		F	0.00	0.473		3.62		1.66
	High-dose	M	4.50	0.657	F: 1.91	5.87	F: 2.30	0.783
		F	4.50	0.903		8.31		0.135

M, male; F, female. Data from low-dose groups were excluded due to concentrations below LLOQ.

In male animals, the high-dose group exhibited 1.6-fold higher C_max_ and 1.5-fold greater AUC_0-last_ than the mid-dose group on D1, increasing to 1.8-fold (C_max_) and 7.0-fold (AUC_0-last_) by D13. In contrast, female animals displayed markedly nonlinear pharmacokinetics: on D1, the high-dose group demonstrated a 32.7-fold higher C_max_ and a 28.2-fold greater AUC_0-last_ compared to the mid-dose group, which normalized to 1.9-fold and 2.3-fold, respectively, by D13.

AF (AUC_0-last,D13_/AUC_0-last,D1_) ranged from 0.135 to 1.66 across sexes and dose groups, indicating no evidence of clinically relevant systemic accumulation. T_max_ varied considerably (0–12 h), reflecting interindividual differences in absorption kinetics.

## 4 Discussion

This study presents the first comprehensive assessment of the safety and TK profiles of PEG IFNα-2b following repeated nebulized inhalation in juvenile SD rats. The findings demonstrate that PEG IFNα-2b, even at a high inhaled dose of 1,202.4 ± 176.4 μg/kg, was well tolerated, with no treatment-related mortality, adverse clinical signs, or histopathological abnormalities. Developmental parameters remained unaffected, and no evidence of systemic or target organ toxicity was observed. Collectively, these results underscore the favorable safety profile of PEG IFNα-2b and support its continued development as an inhaled antiviral therapy for pediatric respiratory infections.

PEGylation is a well-established strategy for improving the pharmacokinetics of protein therapeutics by enhancing molecular stability, prolonging half-life, and reducing immunogenicity (Veronese and Pasut, 2005). In the context of inhalation therapy, PEG IFNα-2b offers additional advantages, such as prolonged pulmonary retention and decreased systemic exposure. In this study, aerosol characterization confirmed that the particle size distribution (MMAD 2.1–2.5 μm; FPF >90%) fall within the optimal particle size range (1–5 μm) for inhaled therapeutics, facilitating efficient deposition in the lower respiratory tract (Gardenhire et al., 2017). Notably, the small particle size is particularly advantageous for pediatric populations, as the narrower airways in neonates and young children necessitate finer aerosols to achieve effective peripheral lung deposition, thereby enhancing therapeutic efficacy. Pulmonary deposition modeling further verified consistent and accurate dose delivery across treatment groups, providing a solid foundation for interpreting downstream toxicological and TK outcomes. TK analyses revealed approximately dose-proportional increases in C_max_ and AUC_0-last_ across mid- and high-dose groups. However, a notable sex difference was observed on D1. The transient elevation in C_max_ and AUC_0-last_ observed in females on D1 may stem from physiological and developmental differences inherent to juvenile animals. This phenomenon is likely multifactorial, involving a combination of developmental, hormonal, and metabolic factors. Firstly, subtle sex-based morphological differences in lung architecture, such as alveolar dimensions and surface area-to-body weight ratios, are established early in development and can influence the efficiency of aerosol deposition and subsequent absorption (Massaro and Massaro, 2006; Saldiva et al., 1995). Secondly, the dosing period of this study coincides with the peripubertal phase in rats, a critical developmental window characterized by dynamic hormonal shifts. Estradiol has been shown to directly regulate the function of alveolar epithelial cells. For example, research has clearly indicated that estradiol can directly activate the epithelial sodium channel (ENaC) *via* the G protein-coupled estrogen receptor (GPER), which is directly related to the dynamic regulation of lung fluid clearance and barrier function (Greenlee et al., 2013). This hormone-mediated direct effect on alveolar epithelial cell function may have temporarily altered the pulmonary absorption microenvironment. Finally, the sex-dependent expression of drug-metabolizing enzymes in the lung cannot be overlooked. The lung tissue itself possesses metabolic capabilities, expressing cytochrome P450 (CYP) isoenzymes. Studies have shown that in multiple organs, including the lungs, the expression of rat CYP enzymes (such as CYP1A2, CYP3A) shows significant sexual dimorphism, with generally higher expression levels in females (Gerges and El-Kadi, 2023). This metabolic difference may have led to a different local clearance rate of PEG IFNα-2b in female rats compared to males, thereby affecting the actual dose entering systemic circulation.

The observed variability in T_max_, ranging from 0 to 12 h, is also likely multifactorial. It may reflect individual differences in inhaled dose retention, mucociliary transport rates, alveolar absorption kinetics, and metabolic degradation of PEG IFNα-2b (Borghardt et al., 2018; Olsson et al., 2011). Furthermore, age-related maturation of pulmonary and systemic circulatory systems in juvenile animals could contribute to inconsistent drug transit times from lung deposition to systemic circulation (Gomes et al., 2001; Jobe, 2015). Importantly, the normalization of exposure by D13 suggests that these differences are transient and unlikely to impact long-term safety or efficacy. The convergence of the PK profiles by D13 is consistent with the rapid physiological maturation during this developmental stage, where initial hormone-driven divergences are compensated by maturing homeostatic and metabolic systems, leading to a more unified drug disposition profile in adulthood.

Interestingly, the body weight gain observed in the PEG IFNα-2b–exposed groups was slightly higher compared to controls, particularly in the mid- and high-dose animals. No clinical or pathological abnormalities were noted. Although most studies report that interferon treatment can lead to weight loss or maintenance rather than weight gain (Quiroga et al., 2019), the weight changes observed in this study remained within acceptable ranges and did not correlate with toxicity. These findings may reflect unique metabolic effects of PEG IFNα-2b in juvenile animals, and further investigation in long-term studies could help clarify the biological relevance of these trends.

Despite the early sex-related differences in systemic exposure, no associated clinical or histopathological abnormalities were observed. This finding is significant, as it indicates that elevated C_max_ and AUC_0-last_ values in females were not biologically consequential. Furthermore, the peak systemic concentrations following inhaled administration remained well below those typically reported with subcutaneous IFNα-2b delivery (Glue et al., 2000), supporting the utility of the inhaled route for achieving effective pulmonary concentrations while minimizing systemic exposure—an essential consideration in pediatric populations where systemic interferon toxicity is a concern.

From a translational standpoint, the establishment of a NOAEL at the highest tested dose (1,202.4 ± 176.4 μg/kg) provides a strong basis for clinical dose selection. Furthermore, the toxicokinetic data, which showed minimal systemic drug accumulation with repeated dosing (AF ranged from 0.135 to 1.66), strongly support the feasibility of an every-other-day administration schedule. This reduced dosing frequency is a significant advantage in pediatrics, where the administration of nebulized therapy can be a distressing and time-consuming procedure for infants and young children, often leading to poor cooperation and adherence (Rau, 2005). When positioned against other inhaled antiviral therapies for respiratory infections, PEG IFNα-2b occupies a unique therapeutic niche. Unlike inhaled ribavirin, whose clinical use is limited by significant safety concerns, including teratogenic risks to caregivers and hematotoxicity in patients (Tejada et al., 2022), the present study has established a high NOAEL for inhaled PEG IFNα-2b, underscoring its favorable safety profile. Compared to highly specific, next-generation approaches like small interfering RNA (siRNA)-based therapies that target viral RNA directly, PEG IFNα-2b acts by stimulating the host’s broad-spectrum innate immune response. While siRNAs have shown promise in clinical trials and may offer a superior safety margin due to their precise targeting, their efficacy is limited to a single, specific pathogen (DeVincenzo et al., 2010). In contrast, the broad-spectrum mechanism of PEG IFNα-2b could be advantageous against a range of respiratory viruses or in cases of viral coinfection, leveraging a well-understood biological pathway combined with an established drug delivery platform.

Despite the strengths of this study, several limitations should be acknowledged. Although juvenile rats are a standard model for pediatric inhalation toxicology, species-specific differences in interferon receptor distribution and downstream signaling pathways may limit the direct extrapolation of findings to human populations (Shaw et al., 2017). Additionally, the 2-week exposure period, while sufficient to assess safety during a critical window of early postnatal development, may not fully capture potential long-term developmental toxicity or very late-onset complications. Future studies involving longer exposure durations and extended observation periods would be valuable to rule out any delayed adverse effects. Furthermore, this study did not include a comprehensive pharmacokinetic assessment across various lung compartments. The inability to quantify systemic exposure in the low-dose group due to concentrations below the LLOQ, while indicating minimal systemic absorption at this dose level, does limit the sensitivity of the TK analysis for defining the lower end of the exposure-response relationship. However, data from a separate investigation have provided valuable insights into the tissue distribution of PEG IFNα-2b (Chu et al., 2025). In that study, following aerosolized inhalation, PEG IFNα-2b exhibited significantly greater drug exposure and prolonged retention in respiratory tissues compared to unmodified IFNα-2b. Specifically, the area under the curve from time zero to time t (AUC_(0–t)_) and mean residence time from time zero to time t (MRT_(0–t)_) in the lungs, trachea, and bronchi were markedly higher in the PEG IFNα-2b group (P < 0.05), with the highest concentrations observed in the lungs. Notably, systemic exposure was negligible, suggesting effective pulmonary targeting. These findings indicate that PEG IFNα-2b may offer pharmacokinetic advantages, such as sustained local drug levels and reduced dosing frequency, which could enhance therapeutic efficacy in respiratory applications and reduce dosing frequency.

Regarding the potential for long-term complications, the current study focused on acute and subacute toxicity endpoints. Within this framework, no histopathological evidence of tissue damage, particularly in the respiratory tract, was observed, which is reassuring for short-term use. Supporting the overall safety profile of PEG IFNα-2b, long-term subcutaneous administration over 48 weeks in patients with chronic hepatitis B has been shown to be well-tolerated, with a low incidence of severe adverse events, such as hematological changes, which were manageable and reversible upon discontinuation (Wang et al., 2024). Although the route of administration differs, these findings from injection therapy provide valuable insights into the compound’s inherent safety characteristics over extended periods, suggesting a favorable baseline for inhaled applications. Potential long-term complications of inhaled biologics could include immunogenic responses or chronic inflammatory changes with prolonged use. While the PEGylation moiety is intended to reduce immunogenicity, and no such signals were detected in this short-term juvenile study, the established safety record from subcutaneous use underscores the potential for minimized risks, though route-specific evaluations remain crucial. The assessment of potential immunogenicity and its long-term consequences remains an important aspect for future clinical monitoring, but the existing data offer a reassuring foundation for further development.

In conclusion, repeated inhaled administration of PEG IFNα-2b in juvenile rats was safe and well tolerated, exhibiting favorable pharmacokinetic properties, negligible systemic accumulation, and no organ-specific toxicity. The transient sex-related differences in early systemic exposure were not associated with adverse effects and diminished with continued dosing. These results support further clinical development of PEG IFNα-2b as a long-acting inhaled antiviral for pediatric respiratory infections. Future studies should focus on confirming local pharmacodynamic activity, optimizing dosing schedules, and evaluating efficacy in viral challenge models and non-human primates to support safe and effective translation to pediatric clinical use.

## 5 Conclusion

This study provides a systematic evaluation of the safety and TK profile of inhaled PEG IFNα-2b in juvenile rats. The compound was well tolerated across all dose levels, with minimal systemic exposure, no evidence of organ-specific toxicity, and no appreciable drug accumulation. Overall, the findings highlight the favorable safety and TK characteristics of PEG IFNα-2b, supporting its continued development as a long-acting, inhaled antiviral therapy for pediatric respiratory infections.

## Data Availability

The original contributions presented in the study are included in the article/Supplementary Material, further inquiries can be directed to the corresponding author.
