# Supplementary material for: Safety evaluation and toxicokinetics of repeated inhalation exposure to pegylated interferon α-2b
Source: Front Pharmacol. 2025 Dec 11;16:1689087. doi: 10.3389/fphar.2025.1689087 (PMC12739654; doi:10.3389/fphar.2025.1689087)
Supplement: Supplementary file 1 [file Supplementaryfile1.docx]

Supplementary Material

# Supplementary Tables

Table S1 Individual Body Weight Data

| **Sex** | **Animal ID** | **Group** | **Weight (g)** | | | | | |
| --- | --- | --- | --- | --- | --- | --- | --- | --- |
|  |  |  | **D-1** | **D4** | **D7** | **D11** | **D13** | **D14** |
| Male | 2474591 | Control | 12 | 21 | 30 | 39 | 43 | 42 |
|  | 2474592 |  | 11 | 19 | 27 | 36 | 40 | 38 |
|  | 2474593 |  | 11 | 19 | 27 | 36 | 41 | 40 |
|  | 2474594 |  | 13 | 22 | 31 | 40 | 44 | 43 |
|  | 2474595 |  | 12 | 20 | 29 | 40 | 44 | 43 |
|  | 2474596 |  | 11 | 19 | 28 | 36 | 42 | 40 |
|  | 2474603 | Low-dose | 13 | 24 | 33 | 44 | 47 | 46 |
|  | 2474604 |  | 11 | 19 | 27 | 35 | 38 | 37 |
|  | 2474605 |  | 12 | 21 | 30 | 40 | 44 | 43 |
|  | 2474606 |  | 13 | 25 | 35 | 43 | 47 | 45 |
|  | 2474607 |  | 12 | 19 | 27 | 35 | 39 | 37 |
|  | 2474608 |  | 12 | 22 | 31 | 43 | 47 | 45 |
|  | 2474615 | Mid-dose | 13 | 22 | 32 | 44 | 49 | 49 |
|  | 2474616 |  | 12 | 21 | 30 | 41 | 45 | 44 |
|  | 2474617 |  | 12 | 23 | 32 | 43 | 49 | 47 |
|  | 2474618 |  | 12 | 22 | 32 | 45 | 50 | 48 |
|  | 2474619 |  | 12 | 21 | 31 | 42 | 47 | 46 |
|  | 2474620 |  | 12 | 22 | 31 | 42 | 48 | 45 |
|  | 2474627 | High-dose | 14 | 26 | 37 | 48 | 53 | 52 |
|  | 2474628 |  | 12 | 22 | 30 | 41 | 46 | 45 |
|  | 2474629 |  | 11 | 20 | 30 | 39 | 44 | 42 |
|  | 2474630 |  | 15 | 27 | 38 | 50 | 56 | 54 |
|  | 2474631 |  | 12 | 21 | 30 | 41 | 46 | 44 |
|  | 2474632 |  | 13 | 23 | 34 | 41 | 45 | 43 |
| Female | 2474597 | Control | 12 | 22 | 30 | 41 | 44 | 42 |
|  | 2474598 |  | 11 | 19 | 27 | 37 | 41 | 39 |
|  | 2474599 |  | 12 | 20 | 30 | 37 | 42 | 40 |
|  | 2474600 |  | 11 | 19 | 28 | 36 | 39 | 36 |
|  | 2474601 |  | 11 | 20 | 28 | 38 | 41 | 40 |
|  | 2474602 |  | 12 | 21 | 28 | 37 | 41 | 39 |
|  | 2474609 | Low-dose | 12 | 23 | 33 | 42 | 47 | 45 |
|  | 2474610 |  | 11 | 18 | 24 | 32 | 35 | 34 |
|  | 2474611 |  | 12 | 22 | 32 | 43 | 49 | 47 |
|  | 2474612 |  | 12 | 23 | 33 | 44 | 47 | 45 |
|  | 2474613 |  | 11 | 17 | 24 | 32 | 35 | 34 |
|  | 2474614 |  | 11 | 21 | 30 | 41 | 44 | 41 |
|  | 2474621 | Mid-dose | 13 | 23 | 33 | 46 | 51 | 49 |
|  | 2474622 |  | 12 | 22 | 31 | 43 | 47 | 45 |
|  | 2474623 |  | 12 | 21 | 30 | 39 | 45 | 42 |
|  | 2474624 |  | 12 | 22 | 32 | 44 | 47 | 46 |
|  | 2474625 |  | 12 | 22 | 31 | 40 | 45 | 43 |
|  | 2474626 |  | 12 | 21 | 29 | 40 | 45 | 43 |
|  | 2474633 | High-dose | 14 | 27 | 37 | 48 | 52 | 50 |
|  | 2474634 |  | 11 | 20 | 29 | 38 | 43 | 41 |
|  | 2474635 |  | 12 | 22 | 31 | 39 | 43 | 42 |
|  | 2474636 |  | 12 | 23 | 34 | 44 | 51 | 48 |
|  | 2474637 |  | 12 | 21 | 30 | 39 | 44 | 42 |
|  | 2474638 |  | 11 | 19 | 28 | 37 | 41 | 39 |

Table S2 Individual Tibial Length Data

| **Sex** | **Animal ID** | **Group** | **Tibial length (mm)** | | |
| --- | --- | --- | --- | --- | --- |
|  |  |  | **D-1** | **D7** | **D13** |
| Male | 2474591 | Control | 15.44 | 24.99 | 30.73 |
|  | 2474592 |  | 15.05 | 21.31 | 28.67 |
|  | 2474593 |  | 15.01 | 22.70 | 30.18 |
|  | 2474603 | Low-dose | 15.51 | 23.99 | 31.12 |
|  | 2474604 |  | 15.35 | 23.34 | 30.18 |
|  | 2474605 |  | 15.63 | 24.19 | 31.63 |
|  | 2474615 | Mid-dose | 15.42 | 23.94 | 30.39 |
|  | 2474616 |  | 15.11 | 23.57 | 31.82 |
|  | 2474617 |  | 15.57 | 23.40 | 31.70 |
|  | 2474627 | High-dose | 15.42 | 25.88 | 31.51 |
|  | 2474628 |  | 15.39 | 23.98 | 29.81 |
|  | 2474629 |  | 15.23 | 23.69 | 30.26 |
| Female | 2474597 | Control | 15.98 | 23.11 | 30.61 |
|  | 2474598 |  | 14.90 | 23.53 | 29.19 |
|  | 2474599 |  | 14.72 | 24.98 | 30.53 |
|  | 2474609 | Low-dose | 15.34 | 23.72 | 29.28 |
|  | 2474610 |  | 14.91 | 22.59 | 29.11 |
|  | 2474611 |  | 15.60 | 24.17 | 30.54 |
|  | 2474621 | Mid-dose | 15.59 | 24.36 | 30.77 |
|  | 2474622 |  | 15.56 | 23.89 | 31.49 |
|  | 2474623 |  | 15.28 | 23.57 | 30.71 |
|  | 2474633 | High-dose | 15.01 | 24.47 | 30.82 |
|  | 2474634 |  | 15.98 | 22.68 | 29.54 |
|  | 2474635 |  | 15.19 | 24.56 | 30.86 |

Table S3 Individual Body Length Data

| **Sex** | **Animal ID** | **Group** | **Body length (mm)** | | |
| --- | --- | --- | --- | --- | --- |
|  |  |  | **D-1** | **D7** | **D13** |
| Male | 2474591 | Control | 57.08 | 62.26 | 80.10 |
|  | 2474592 |  | 55.21 | 61.67 | 79.92 |
|  | 2474593 |  | 54.20 | 64.00 | 79.78 |
|  | 2474603 | Low-dose | 55.52 | 60.42 | 85.35 |
|  | 2474604 |  | 54.87 | 60.70 | 79.23 |
|  | 2474605 |  | 54.39 | 61.88 | 80.78 |
|  | 2474615 | Mid-dose | 55.62 | 66.84 | 81.41 |
|  | 2474616 |  | 54.52 | 64.23 | 83.25 |
|  | 2474617 |  | 53.58 | 62.61 | 82.40 |
|  | 2474627 | High-dose | 55.70 | 67.79 | 86.13 |
|  | 2474628 |  | 55.67 | 62.62 | 82.32 |
|  | 2474629 |  | 54.78 | 65.88 | 81.51 |
| Female | 2474597 | Control | 55.64 | 60.31 | 79.52 |
|  | 2474598 |  | 54.90 | 62.20 | 80.48 |
|  | 2474599 |  | 54.13 | 66.00 | 81.71 |
|  | 2474609 | Low-dose | 54.38 | 63.38 | 80.23 |
|  | 2474610 |  | 54.42 | 59.23 | 80.42 |
|  | 2474611 |  | 54.14 | 66.95 | 82.22 |
|  | 2474621 | Mid-dose | 54.89 | 65.77 | 85.88 |
|  | 2474622 |  | 55.63 | 63.61 | 83.07 |
|  | 2474623 |  | 55.01 | 63.47 | 79.81 |
|  | 2474633 | High-dose | 55.69 | 64.40 | 84.75 |
|  | 2474634 |  | 55.46 | 63.09 | 81.80 |
|  | 2474635 |  | 55.13 | 65.82 | 80.85 |

Table S4 Individual Tail Length Data

| **Sex** | **Animal ID** | **Group** | **Tail length (mm)** | | |
| --- | --- | --- | --- | --- | --- |
|  |  |  | **D-1** | **D7** | **D13** |
| Male | 2474591 | Control | 23.72 | 40.39 | 57.57 |
|  | 2474592 |  | 24.10 | 40.50 | 55.73 |
|  | 2474593 |  | 24.00 | 39.79 | 57.14 |
|  | 2474603 | Low-dose | 25.67 | 46.55 | 61.26 |
|  | 2474604 |  | 25.15 | 41.11 | 59.15 |
|  | 2474605 |  | 25.15 | 43.69 | 61.64 |
|  | 2474615 | Mid-dose | 24.41 | 44.10 | 59.48 |
|  | 2474616 |  | 24.27 | 44.51 | 63.09 |
|  | 2474617 |  | 25.80 | 41.38 | 62.40 |
|  | 2474627 | High-dose | 23.78 | 44.27 | 60.68 |
|  | 2474628 |  | 25.36 | 43.08 | 60.92 |
|  | 2474629 |  | 24.18 | 43.02 | 59.24 |
| Female | 2474597 | Control | 25.79 | 46.00 | 61.14 |
|  | 2474598 |  | 22.14 | 40.01 | 56.61 |
|  | 2474599 |  | 23.66 | 44.11 | 59.53 |
|  | 2474609 | Low-dose | 25.17 | 41.42 | 58.35 |
|  | 2474610 |  | 24.20 | 40.16 | 58.70 |
|  | 2474611 |  | 26.06 | 46.82 | 68.52 |
|  | 2474621 | Mid-dose | 25.06 | 43.91 | 63.18 |
|  | 2474622 |  | 24.18 | 43.96 | 64.65 |
|  | 2474623 |  | 25.38 | 43.70 | 58.43 |
|  | 2474633 | High-dose | 25.27 | 44.38 | 64.34 |
|  | 2474634 |  | 25.63 | 42.72 | 61.99 |
|  | 2474635 |  | 25.13 | 44.05 | 66.41 |

Table S5 Hematological Analysis at D14

| **Parameter** | **Sex** | **Control** | **Low-dose** | **Mid-dose** | **High-dose** |
| --- | --- | --- | --- | --- | --- |
| **WBC**  **(10^9^/L)** | Male | 2.027 ± 0.936 | 2.230 ± 0.791 | 2.233 ± 1.053 | 1.737 ± 0.489 |
|  | Female | 1.830 ± 0.287 | 2.153 ± 1.102 | 2.320 ± 0.792 | 2.037 ± 0.434 |
| **Neut**  **(10^9^/L)** | Male | 0.243 ± 0.129 | 0.333 ± 0.065 | 0.323 ± 0.166 | 0.433 ± 0.127 |
|  | Female | 0.257 ± 0.015 | 0.383 ± 0.212 | 0.410 ± 0.234 | 0.307 ± 0.035 |
| **Neut**  **(%)** | Male | 12.40 ± 3.61 | 15.93 ± 4.47 | 14.67 ± 4.14 | **24.97 ± 2.65*** |
|  | Female | 14.30 ± 2.77 | 18.33 ± 3.94 | 16.87 ± 3.67 | 15.63 ± 4.28 |
| **Lymph**  **(10^9^/L)** | Male | 1.590 ± 0.824 | 1.617 ± 0.672 | 1.660 ± 0.819 | 1.130 ± 0.308 |
|  | Female | 1.313 ± 0.283 | 1.530 ± 0.821 | 1.617 ± 0.487 | 1.470 ± 0.422 |
| **Lymph**  **(%)** | Male | 76.20 ± 8.37 | 71.50 ± 4.34 | 74.13 ± 4.14 | 65.23 ± 4.97 |
|  | Female | 71.37 ± 4.24 | 69.77 ± 4.81 | 70.23 ± 3.01 | 71.47 ± 5.60 |
| **Mono**  **(10^9^/L)** | Male | 0.187 ± 0.012 | 0.263 ± 0.085 | 0.230 ± 0.111 | 0.167 ± 0.065 |
|  | Female | 0.247 ± 0.040 | 0.227 ± 0.085 | 0.277 ± 0.064 | 0.250 ± 0.066 |
| **Mono**  **(%)** | Male | 11.13 ± 6.24 | 11.93 ± 1.27 | 10.30 ± 0.30 | 9.50 ± 2.29 |
|  | Female | 13.57 ± 2.11 | 11.43 ± 2.85 | 12.27 ± 1.95 | 12.40 ± 3.05 |
| **Eos**  **(10^9^/L)** | Male | 0.007 ± 0.006 | 0.010 ± 0.010 | 0.013 ± 0.006 | 0.007 ± 0.012 |
|  | Female | 0.007 ± 0.006 | 0.007 ± 0.006 | 0.010 ± 0.010 | 0.007 ± 0.006 |
| **Eos**  **(%)** | Male | 0.27 ± 0.23 | 0.40 ± 0.46 | 0.67 ± 0.32 | 0.30 ± 0.52 |
|  | Female | 0.40 ± 0.35 | 0.23 ± 0.21 | 0.37 ± 0.32 | 0.30 ± 0.26 |
| **Baso**  **(10^9^/L)** | Male | 0.000 ± 0.000 | 0.007 ± 0.012 | 0.007 ± 0.006 | 0.000 ± 0.000 |
|  | Female | 0.007 ± 0.006 | 0.007 ± 0.012 | 0.007 ± 0.006 | 0.003 ± 0.006 |
| **Baso**  **(%)** | Male | 0.00 ± 0.00 | 0.23 ± 0.40 | 0.23 ± 0.21 | 0.00 ± 0.00 |
|  | Female | 0.37 ± 0.32 | 0.23 ± 0.40 | 0.27 ± 0.25 | 0.20 ± 0.35 |
| **RBC**  **(10^12^/L)** | Male | 3.903 ± 0.452 | 3.857 ± 0.127 | 4.003 ± 0.140 | 3.953 ± 0.180 |
|  | Female | 3.767 ± 0.133 | 3.810 ± 0.148 | 3.917 ± 0.197 | 3.900 ± 0.316 |
| **HGB**  **(g/L)** | Male | 73.3 ± 6.7 | 72.0 ± 4.4 | 75.3 ± 3.5 | 71.7 ± 13.8 |
|  | Female | 75.0 ± 2.6 | 79.0 ± 1.7 | 79.0 ± 1.7 | 70.0 ± 16.1 |
| **HCT**  **(%)** | Male | 23.50 ± 2.43 | 22.77 ± 1.21 | 23.70 ± 0.72 | 22.63 ± 3.72 |
|  | Female | 23.37 ± 0.91 | 24.23 ± 0.32 | 24.60 ± 1.25 | 22.00 ± 4.42 |
| **Retic**  **(%)** | Male | 15.207 ± 2.287 | 13.190 ± 2.540 | 15.433 ± 1.607 | 17.713 ± 3.959 |
|  | Female | 15.580 ± 0.306 | 12.303 ± 2.558 | 13.760 ± 1.089 | 17.187 ± 6.105 |
| **MCV**  **(fL)** | Male | 60.27 ± 1.08 | 59.03 ± 1.54 | 59.23 ± 2.21 | 57.10 ± 7.73 |
|  | Female | 62.03 ± 0.32 | 63.63 ± 1.59 | 62.80 ± 1.32 | 56.10 ± 7.87 |
| **MCHC**  **(g/L)** | Male | 312.3 ± 3.8 | 316.3 ± 9.0 | 317.7 ± 5.1 | 315.7 ± 10.2 |
|  | Female | 321.0 ± 7.0 | 326.0 ± 9.5 | 321.3 ± 10.3 | 316.7 ± 10.3 |
| **MCH**  **(pg)** | Male | 18.83 ± 0.50 | 18.67 ± 0.90 | 18.83 ± 0.81 | 18.07 ± 2.94 |
|  | Female | 19.93 ± 0.35 | 20.77 ± 1.07 | 20.17 ± 0.78 | 17.83 ± 3.07 |
| **PLT**  **(10^9^/L)** | Male | 978.7 ± 21.5 | 843.0 ± 79.0 | 888.3 ± 88.6 | 880.3 ± 121.9 |
|  | Female | 909.7 ± 137.5 | 781.3 ± 67.1 | 869.0 ± 66.0 | 876.0 ± 169.3 |
| **Retic**  **(10^12^/L)** | Male | 0.59430 ± 0.11291 | 0.50910 ± 0.10418 | 0.61690 ± 0.05229 | 0.69717 ± 0.13431 |
|  | Female | 0.58660 ± 0.01049 | 0.47040 ± 0.10947 | 0.53757 ± 0.01807 | 0.65923 ± 0.18538 |

WBC, white blood cell count; Neut, neutrophil count; Lymph, lymphocyte count; Mono, monocyte count; Eos, eosinophil count; Baso, basophil count; RBC, red blood cell count; HGB, hemoglobin; HCT, hematocrit; Retic, reticulocyte count; MCV, mean corpuscular volume; MCH, mean corpuscular hemoglobin; MCHC, mean corpuscular hemoglobin concentration; PLT, platelet count. Data are shown as Mean ± SD. *P < 0.05 vs. control by ANOVA & Dunnett test.

Table S6 Coagulation Parameters at D14

| **Parameter** | **Sex** | **Control** | **Low-dose** | **Mid-dose** | **High-dose** |
| --- | --- | --- | --- | --- | --- |
| **PT (s)** | Male | 7.13 ± 0.12 | 7.13 ± 0.06 | 7.17 ± 0.15 | 7.20 ± 0.00 |
|  | Female | 7.33 ± 0.06 | 7.13 ± 0.06 | 7.27 ± 0.23 | 7.13 ± 0.06 |
| **APTT (s)** | Male | 11.40 ± 0.00 | 11.50 ± 0.00 | 11.50 ± 0.00 | 11.55 ± 0.07 |
|  | Female | 11.47 ± 0.06 | 11.43 ± 0.06 | 11.50 ± 0.10 | 11.50 ± 0.10 |
| **FIB (g/L)** | Male | 1.7317 ± 0.1666 | 1.6317 ± 0.1327 | 1.7917 ± 0.1009 | 1.9880 ± 0.1696 |
|  | Female | 1.6733 ± 0.0745 | 1.7280 ± 0.2705 | 1.7753 ± 0.0533 | 1.7733 ± 0.1220 |

PT, prothrombin time; APTT, activated partial thromboplastin time; FIB, fibrinogen. Data are shown as Mean ± SD.

Table S7 Serum Biochemistry Profiles at D14

| **Parameter** | **Sex** | **Control** | **Low-dose** | **Mid-dose** | **High-dose** |
| --- | --- | --- | --- | --- | --- |
| **ALT**  **(U/L)** | Male | 29.3 ± 5.5 | 29.3 ± 2.9 | 24.7 ± 3.1 | 31.3 ± 6.5 |
|  | Female | 25.00 ± 1.00 | 21.00 ± 0.70 | 21.30 ± 0.70 | 21.30 ± 1.70 |
| **AST**  **(U/L)** | Male | 150.7 ± 10.8 | 146.3 ± 22.9 | 148.3 ± 10.0 | 138.3 ± 17.8 |
|  | Female | 144.30 ± 7.50 | 138.30 ± 23.10 | 126.00 ± 8.70 | 140.00 ± 17.70 |
| **ALP**  **(U/L)** | Male | 332.7 ± 29.7 | 332.0 ± 78.4 | 356.0 ± 59.0 | 330.7 ± 11.0 |
|  | Female | 366.70 ± 38.60 | 315.70 ± 40.70 | 287.70 ± 26.80 | 349.30 ± 61.40 |
| **TBil (µmol/L)** | Male | 3.823 ± 0.825 | 3.723 ± 0.818 | 3.603 ± 0.245 | 3.807 ± 0.248 |
|  | Female | 4.15 ± 0.40 | 4.01 ± 0.31 | 3.70 ± 0.04 | 3.95 ± 0.40 |
| **DBil (µmol/L)** | Male | 1.260 ± 0.396 | 1.290 ± 0.396 | 1.147 ± 0.175 | 1.200 ± 0.052 |
|  | Female | 1.29 ± 0.18 | 1.49 ± 0.23 | 1.00 ± 0.05 | 1.26 ± 0.33 |
| **GGT**  **(U/L)** | Male | 1.0 ± 0.0 | **0.0 ± 0.0*** | 1.7 ± 0.6 | 1.0 ± 0.0 |
|  | Female | 1.00 ± 0.00 | 0.70 ± 0.60 | 0.70 ± 0.60 | 1.70 ± 1.00 |
| **TP**  **(g/L)** | Male | 48.27 ± 2.58 | 46.83 ± 0.40 | 49.23 ± 1.79 | 49.33 ± 0.68 |
|  | Female | 48.77 ± 2.48 | 46.90 ± 1.83 | 49.00 ± 1.55 | 48.67 ± 1.86 |
| **Alb**  **(g/L)** | Male | 28.07 ± 0.91 | 27.63 ± 0.65 | 28.70 ± 0.36 | 28.90 ± 0.89 |
|  | Female | 28.50 ± 0.89 | 27.70 ± 0.61 | 28.80 ± 0.52 | 28.53 ± 1.07 |
| **A/G** | Male | 1.40 ± 0.10 | 1.43 ± 0.15 | 1.40 ± 0.10 | 1.40 ± 0.10 |
|  | Female | 1.40 ± 0.10 | 1.47 ± 0.06 | 1.43 ± 0.15 | 1.43 ± 0.06 |
| **Glu**  **(mmol/L)** | Male | 5.917 ± 0.287 | **4.950 ± 0.322*** | 5.503 ± 0.325 | 6.087 ± 0.150 |
|  | Female | 6.37 ± 0.52 | 6.48 ± 0.72 | 6.58 ± 0.32 | 7.04 ± 0.74 |
| **UREA**  **(mmol/L)** | Male | 4.87 ± 0.55 | 5.10 ± 0.96 | 3.63 ± 0.35 | 5.23 ± 0.78 |
|  | Female | 3.97 ± 0.21 | 3.50 ± 0.17 | 3.07 ± 0.47 | 3.93 ± 0.75 |
| **Cre**  **(mmol/L)** | Male | 14.3 ± 1.5 | 15.7 ± 0.6 | 15.7 ± 1.2 | 14.7 ± 1.2 |
|  | Female | 14.00 ± 1.00 | 15.00 ± 1.00 | 14.30 ± 1.50 | 15.00 ± 1.00 |
| **CK**  **(U/L)** | Male | 447.7 ± 18.0 | 1042.7 ± 963.4 | 519.7 ± 70.2 | 447.0 ± 112.5 |
|  | Female | 422.70 ± 19.60 | 413.00 ± 129.70 | 394.00 ± 114.80 | 416.00 ± 35.50 |
| **LDH**  **(U/L)** | Male | 1000.0 ± 99.3 | 742.3 ± 234.0 | 1189.0 ± 148.0 | 1000.3 ± 340.8 |
|  | Female | 606.70 ± 107.80 | 698.70 ± 416.30 | 744.00 ± 350.70 | 863.30 ± 182.70 |
| **TC**  **(mmol/L)** | Male | 3.667 ± 1.146 | 3.663 ± 0.221 | 3.700 ± 0.432 | 3.787 ± 0.597 |
|  | Female | 4.80 ± 0.95 | 3.88 ± 0.77 | 3.78 ± 0.56 | 4.23 ± 0.12 |
| **TG**  **(mmol/L)** | Male | 0.880 ± 0.202 | 0.650 ± 0.260 | 0.787 ± 0.301 | 1.407 ± 0.594 |
|  | Female | 0.56 ± 0.11 | 0.37 ± 0.07 | 0.53 ± 0.11 | 0.69 ± 0.19 |
| **Ca**  **(mmol/L)** | Male | 2.613 ± 0.104 | 2.617 ± 0.049 | 2.603 ± 0.047 | 2.657 ± 0.091 |
|  | Female | 2.61 ± 0.07 | 2.56 ± 0.01 | 2.64 ± 0.09 | 2.59 ± 0.02 |
| **P**  **(mmol/L)** | Male | 3.257 ± 0.162 | 3.157 ± 0.070 | 3.183 ± 0.086 | 3.117 ± 0.137 |
|  | Female | 3.19 ± 0.15 | 3.13 ± 0.12 | 3.11 ± 0.15 | 3.07 ± 0.07 |
| **Na⁺**  **(mmol/L)** | Male | 137.53 ± 0.91 | 136.37 ± 0.90 | 137.17 ± 0.51 | 136.50 ± 0.79 |
|  | Female | 137.53 ± 0.83 | 137.60 ± 0.35 | 136.93 ± 0.42 | 136.73 ± 0.12 |
| **K⁺**  **(mmol/L)** | Male | 5.403 ± 0.108 | 5.360 ± 0.339 | 5.283 ± 0.167 | 5.233 ± 0.240 |
|  | Female | 4.97 ± 0.11 | 5.20 ± 0.41 | 5.19 ± 0.23 | 5.18 ± 0.27 |
| **Cl⁻**  **(mmol/L)** | Male | 104.80 ± 0.26 | 103.23 ± 0.29 | 103.57 ± 0.90 | 104.07 ± 2.65 |
|  | Female | 103.20 ± 0.95 | 104.20 ± 1.51 | 104.50 ± 0.36 | 103.87 ± 0.45 |

ALT, alanine aminotransferase; AST, aspartate aminotransferase; ALP, alkaline phosphatase; TBil, total bilirubin; DBil, direct bilirubin; GGT, γ-glutamyl transferase; TP, total protein; Alb, albumin; A/G, albumin/globulin ratio; Glu, glucose; UREA, urea; Cre, creatinine; CK, creatine kinase; LDH, lactate dehydrogenase; TC, total cholesterol; TG, triglyceride; Ca, calcium; P, phosphorus; Na, sodium; K, potassium; Cl, chloride. Data are shown as Mean ± SD. *P < 0.05 vs. control by ANOVA & Dunnett test or Kruskal-Wallis & Wilcoxon test.

# Supplementary Figures


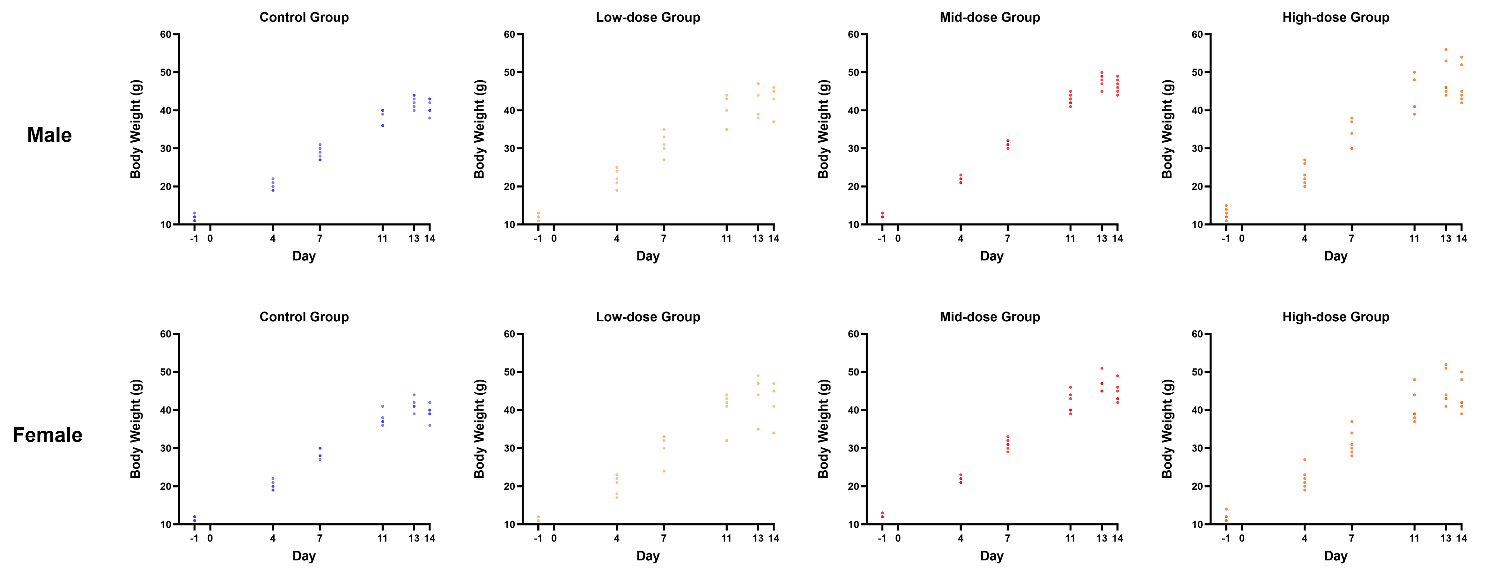


Figure S1 Scatter plot of individual body weight data


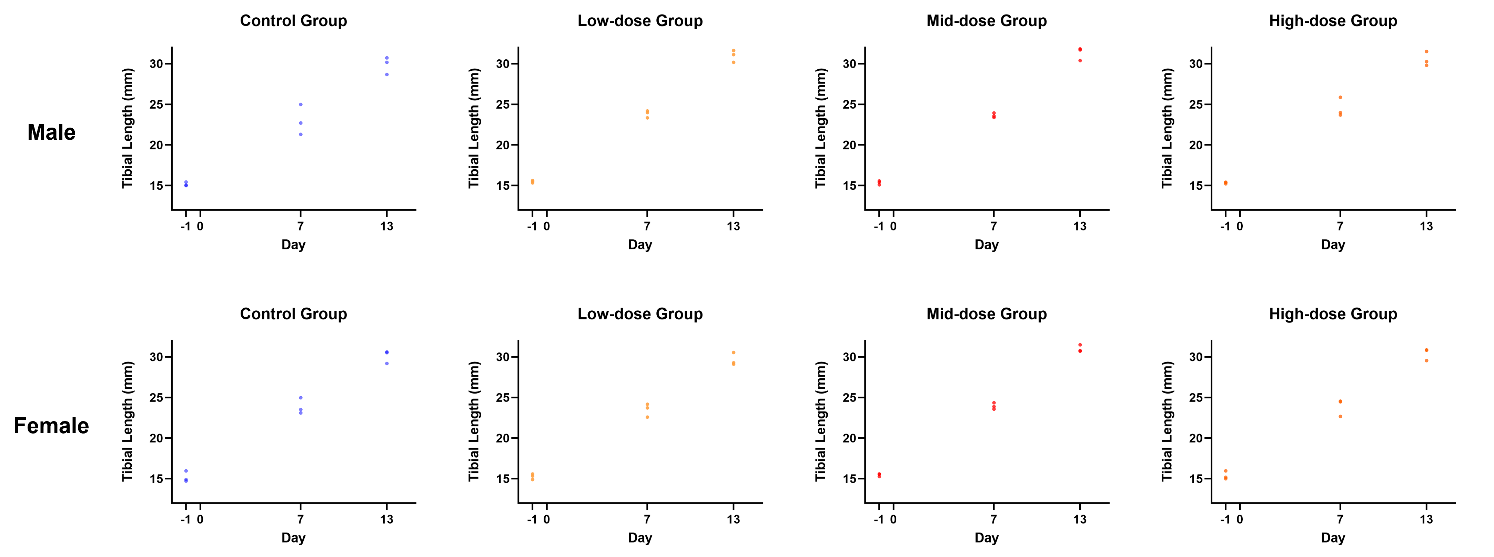


Figure S2 Scatter plot of individual tibial length data


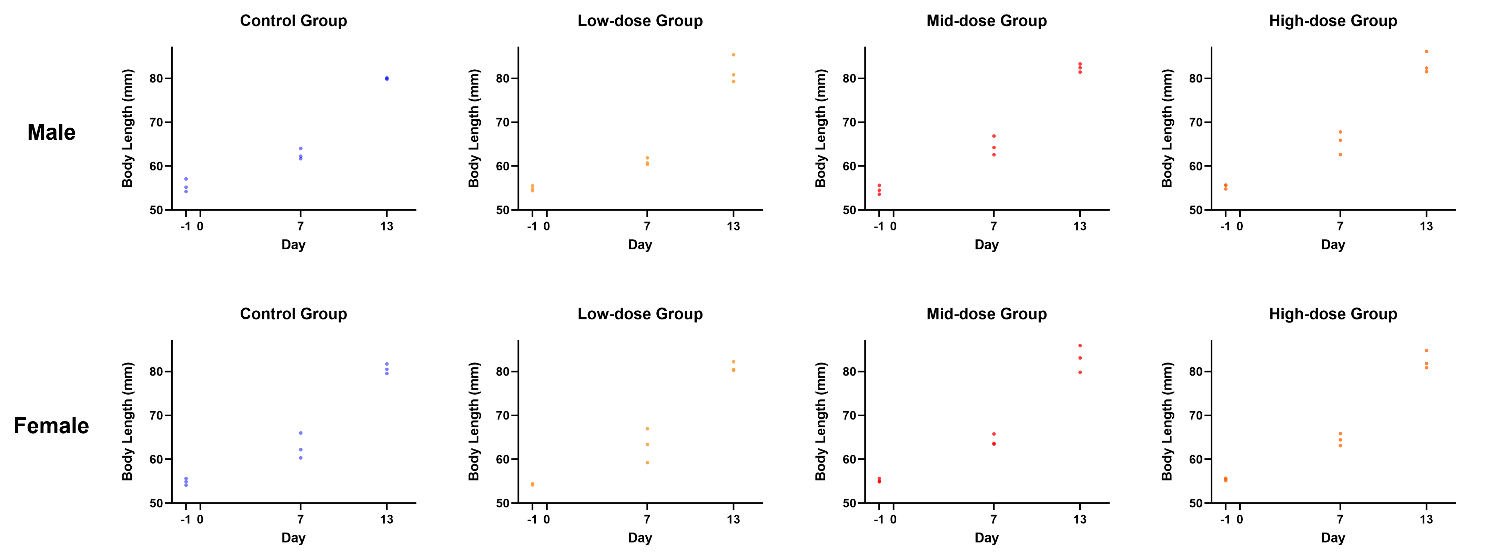


Figure S3 Scatter plot of individual body length data


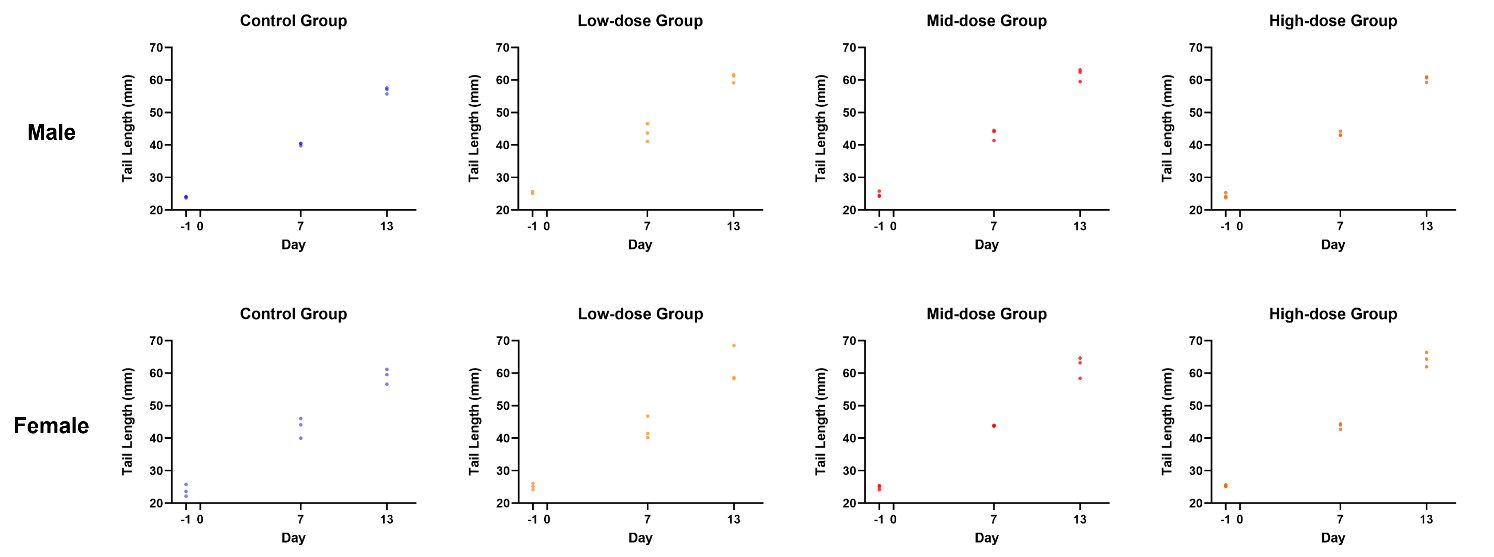


Figure S4 Scatter plot of individual tail length data
